# Harnessing the immunomodulatory properties of HLA-G for advanced immunotherapies

**DOI:** 10.3389/fimmu.2025.1716778

**Published:** 2025-12-09

**Authors:** Xeni Beli, Nikolaos Savvopoulos, Dionysia Kefala, Memnon Lysandrou, Maria Liga, Alexandros Spyridonidis

**Affiliations:** 1 Bone Marrow Transplantation Unit and Institute of Cell Therapy, University of Patras, Rio, Greece; 2 Department of Hematology, Amsterdam University Medical Center (UMC), Vrije Universiteit Amsterdam, Amsterdam, Netherlands; 3 Department of Medicine I, Medical Center, University of Freiburg, Freiburg, Germany

**Keywords:** human leukocyte antigen-G (HLA-G), graft versus host disease (GVHD), adoptive cellular immunotherapy, immune tolerance, tumor immune escape, immune checkpoint

## Abstract

Human leukocyte antigen-G (HLA-G) is a non-classical MHC class I molecule with unique immunomodulatory properties that extend far beyond its well-established role at the maternal-fetal interface. Increasing evidence highlights HLA-G as a pivotal regulator of immune homeostasis, capable of shaping both innate and adaptive cytotoxic responses. It exerts a context-dependent role, promoting tolerance in settings such as transplantation and autoimmunity while contributing to immune evasion in cancer and infection, and this functional plasticity is further shaped by its isoform diversity and its interplay with other non-classical HLA class I molecules. In this review, we discuss key aspects of HLA-G biology, particularly its capacity to promote immune tolerance or facilitate immune escape, and how these insights can be leveraged in the development of cellular and acellular immunotherapies. We further summarize current strategies that incorporate or target HLA-G in the treatment of malignancies and autoimmune diseases, and highlight its emerging potential as a therapeutic target.

## Introduction

Among non-classical MHC class I molecules, HLA-G stands out for its unique structural and functional features. Initially discovered at the maternal-fetal interface, HLA-G was shown to be indispensable for protecting the semi-allogeneic fetus from maternal immune rejection (1, 2). This early finding framed HLA-G as a tolerogenic molecule, but subsequent studies revealed that its influence extends far beyond reproduction, encompassing tumor biology, transplantation, infection, and autoimmunity. However, accumulating evidence indicates that the functional outcome of HLA-G expression is highly context-dependent, with heterogeneous expression patterns across tissues and disease states, making its clinical interpretation particularly challenging. In each of these settings, HLA-G acts as a regulator of cytotoxic effector functions, positioning it as a unique immune checkpoint of growing translational relevance, among other non-classical HLA class I molecules such as HLA-E and HLA-F.

Physiological expression of HLA-G is largely restricted to pregnancy, particularly at the maternal-fetal interface, where it establishes an immune-protective niche. Although HLA-G is epigenetically silenced in most adult tissues due to promoter methylation, its expression has been detected in immune-privileged sites such as the thymus and the cornea. Low frequencies of HLA-G^+^ immune cells have also been identified in the peripheral blood of healthy individuals, confined to small, tightly regulated subsets of T cells, Natural Killer (NK) cells and monocytes, representing a controlled, homeostatic immunoregulatory mechanism (3, 4). In pathological contexts-including malignancy, infection, and autoimmunity-ectopic HLA-G expression is frequently observed and has been associated with disease progression, underscoring its relevance as a prognostic biomarker as well as a mediator of immune tolerance beyond its role in maternal-fetal tolerance (5).

Consistent with its diverse immunoregulatory functions in different contexts, HLA-G displays remarkable structural variability. Through alternative splicing, at least seven isoforms are generated including the membrane-bound (HLA-G1-4) and the secreted soluble forms (HLA-G5-7), indicating high diversity (6). HLA-G1 is the only full-length, membrane-bound and β2-microglobulin (β2m)-associated form, while its soluble counterpart HLA-G5 also binds β2m. In contrast, HLA-G2, G3, G4, G6 and G7 lack one or more extracellular domains (α2 and/or α3), do not associate with β2m, and are therefore structurally truncated (7). Functionally, HLA-G exerts its effects primarily by dampening the cytotoxic activity of immune effector cells. Membrane-bound isoforms primarily act through direct cell-cell interactions, delivering inhibitory signals to both NK cell- and T cell-mediated cytolysis (8), while soluble HLA-G has been shown to trigger apoptosis in activated CD8^+^ T cells and suppress both NK cell activity and allo-reactive cytotoxic T lymphocyte responses (9). Membrane-bound HLA-G1 can also be released into the circulation through proteolytic shedding (10). By engaging inhibitory receptors on NK cells and cytotoxic T lymphocytes (CTLs), HLA-G suppresses their ability to lyse target cells and to secrete effector cytokines such as IFN-γ and TNF-α and thus regulate cytotoxic effector functions. This suppressive capacity is indispensable for establishing immune protection in pregnancy and transplantation, however in tumors and infections the same mechanism is co-opted to enable immune evasion. HLA-G mediates its immunomodulatory functions primarily through the inhibitory Immunoglobulin-like Transcript (ILT) 2 on NK, T, B and some myeloid cells, ILT4 on monocytes, macrophages and dendritic cells, and KIR2DL4 mainly on NK and a subset of CD8^+^ T cells. This receptor diversity ensures that HLA-G is capable of shaping the activity of multiple innate and adaptive immune populations (11).

Complementing HLA-G, the non-classical HLA molecule HLA-E suppresses NK and CD8^+^ T-cell activity through engagement of the inhibitory receptor NKG2A/CD94, and its overexpression has been associated with poor clinical outcomes in several malignancies placing it as a potential biomarker (12). Therapeutic blockade of this axis is therefore being explored to restore antitumor immunity (13). In addition, HLA-F, expressed on activated or virus-infected cells as well as extravillous trophoblasts, interacts with receptors such as KIR3DS1/L1, suggesting potential roles in immune evasion (14). Together, these molecules form an integrated network of non-classical HLA-mediated immune checkpoints, with HLA-G at the center and HLA-E/HLA-F providing complementary mechanisms of immune modulation relevant to both tolerance and tumor escape (15, 16).

Taken together, this mini-review focuses on HLA-G at the intersection of immune regulation and immune escape. We first discuss how HLA-G function contributes to a more tolerogenic environment, either in physiological or pathological contexts. We then explore how HLA-G modulates cytotoxic mechanisms, particularly the activity of NK cells and CTLs in tumor microenvironment. Finally, we highlight emerging strategies that harness or block HLA-G in therapeutic contexts, positioning this non-classical HLA molecule as a next-generation checkpoint for advanced immunotherapies.

## HLA-G as a regulator of immune tolerance

**Figure 1:**
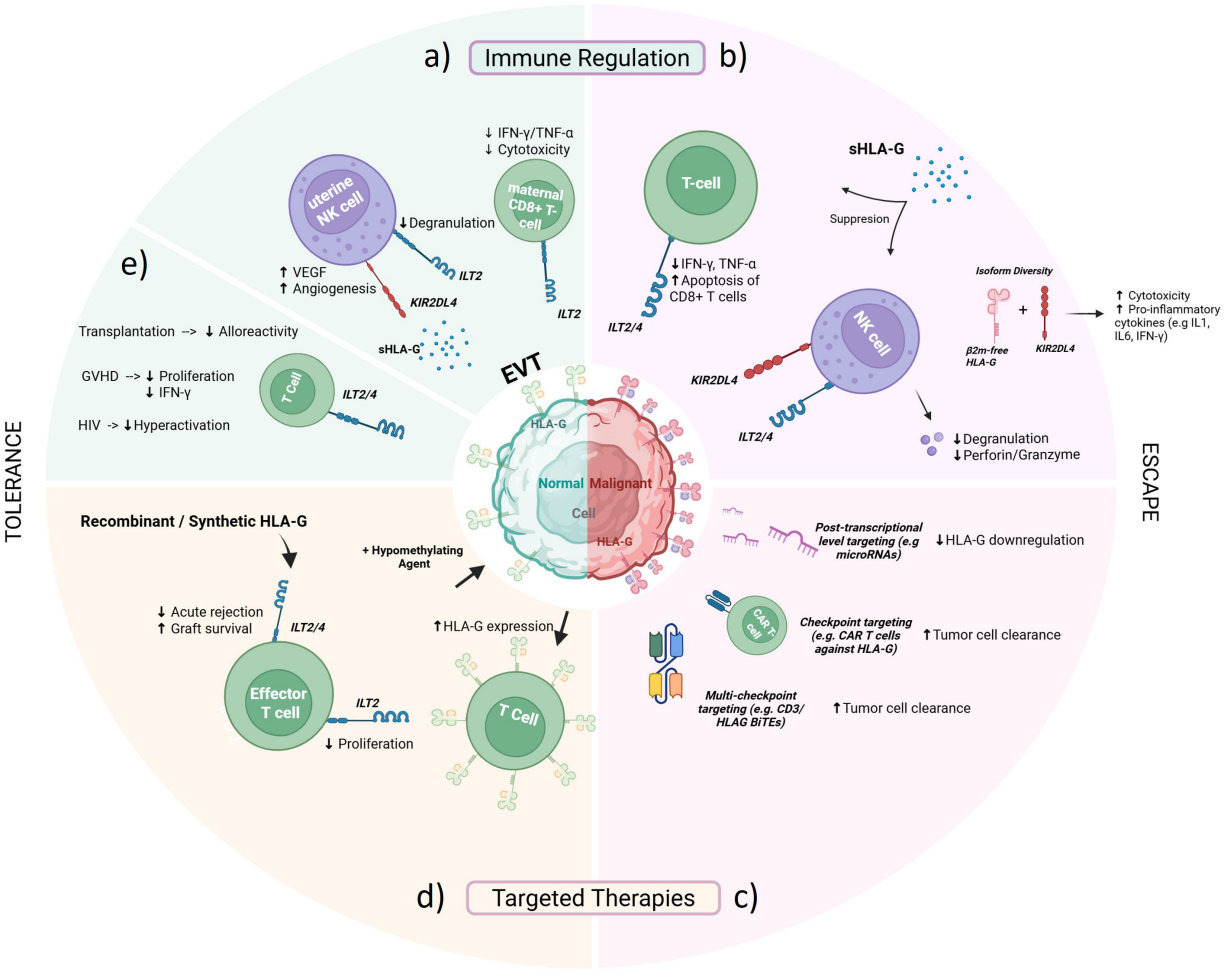
Schematic overview of the context-dependent role of HLA-G and emerging therapies. **(a)** HLA-G–mediated immune tolerance in pregnancy, highlighting its pivotal role in establishing fetal-maternal tolerance, promoting vascular remodeling and modulating immune responses through interactions with ILT2 and KIR2DL4 receptors on T and NK cells. **(b)** Tumor immune evasion mediated by aberrant or ectopic HLA-G expression, leading to suppression of cytotoxic T and NK cell functions and facilitation of tumor growth, metastasis, and immune escape. **(c, d)** Novel therapeutic strategies harnessing or counteracting the regulatory properties of HLA-G, including recombinant or synthetic HLA-G to promote tolerance in transplantation settings, hypomethylating agents to modulate HLA-G expression, and immune checkpoint–based or CAR-T-mediated approaches targeting HLA-G to restore anti-tumor immunity and improve tumor clearance. **(e)** Protective regulatory function of HLA-G in pathological conditions, such as transplantation, graft-versus-host disease, and HIV infection (ILT, Immunoglobulin-like Transcript; KIR2DL4, Killer Cell Immunoglobulin like Receptor 2DL4; β2m, beta-2 microglobulin; sHLA-G, soluble HLA-G; VEGF, Vascular Endothelial Growth Factor; GvHD, Graft versus Host Disease) (Created in https://BioRender.com).

HLA-G has emerged as a key tolerogenic molecule with critical functions in both physiological and pathological settings. The primary study by Kovats et al. showed that extravillous trophoblasts express HLA-G which interacts with inhibitory receptors on maternal NK cells to prevent cytotoxicity against placental cells (1). Direct functional evidence further demonstrated that HLA-G expression on cytotrophoblasts protect placental cells from NK mediated cytotoxicity (2). Additionally, engagement of the NK cell receptor KIR2DL4 by soluble HLA-G secreted at the maternal-fetal interface triggers the release of pro-angiogenic cytokines and support vascular remodeling, a process essential for placental development, establishment of maternal-fetal circulation, and successful maintenance of pregnancy (**Figure 1a**) (17). HLA-G expression has also been observed in immune cells of healthy individuals. For instance, a naturally occurring population of CD14^+^HLA-G^+^HLA-DR^low^ monocytes was identified in peripheral blood, where they suppressed T-cell proliferation and IFN-γ production, further supporting a regulatory role for HLA-G in maintaining immune homeostasis (18).

HLA-G has also been studied in the context of pathological conditions (**Figure 1e**). In 2007, a distinct subset of HLA-G^+^ regulatory T cells was identified in human peripheral blood and sites of inflammation with strong FOXP3-independent immunosuppressive properties (3). Furthermore, following allogeneic Hematopoietic Stem Cell Transplantation (HSCT), the frequency of HLA-G^+^ cells in blood markedly increases. Notably, besides an increase in CD14^+^HLA-G^+^ cells, there was also a pronounced expansion of CD3^+^HLA-G^+^ cells (18). Hypomethylation at CpG cites within the 5’UTR of the HLA-G gene, correlates with vascular calcification scores, thereby linking epigenetic regulation of HLA-G to the severity of coronary heart disease (19). Another study reported that high levels of HLA-G in allogeneic umbilical cord blood-derived hematopoietic stem cells protected them against NK cytolysis, supporting its role as a natural mediator of graft acceptance (20). In HIV, HLA-G appears to play a beneficial role by dampening excessive activation. A population of CD4^+^HLA-G^+^ T cells that is preserved in long-term non progressors has been identified and shown to mitigate immune hyperactivation (21). Further supporting these findings, in immune thrombocytopenia, reduced soluble and membrane HLA-G expression, along with decreased ILT2/ILT4 receptors levels on CD4^+^ T cells and monocytes has been reported and upon treatment with recombinant HLA-G, ILT expression was restored and cytokine production shifted toward an anti-inflammatory profile (22). Collectively, these findings highlight the relevance of HLA-G as an immunoregulatory molecule across both physiological and disease-associated conditions.

## HLA-G as a mediator of immune escape

The tolerogenic features that ensure maternal-fetal immune success were subsequently found to have profound implications for tumor biology, transplantation, and infection, with HLA-G emerging as a central regulator of cytotoxic effector functions across these settings. A central theme that has emerged is the ability of HLA-G to shape the effector arm of immunity by modulating the cytotoxic potential of both innate and adaptive lymphocytes. In this respect, HLA-G has evolved from being viewed as a passive tolerogenic marker into an active immune checkpoint whose expression status directly informs the cytotoxic capacity of NK cells and cytotoxic T lymphocytes (CTLs).

The first direct demonstration that HLA-G suppresses cytotoxicity came from the seminal observation that the α1 domain of HLA-G1 and HLA-G2 was sufficient to inhibit NK cell-mediated lysis of target cell (23). These findings were reinforced when soluble isoforms of HLA-G were purified and shown to inhibit NK cytolysis *in vitro* (24). The idea that HLA-G might serve as a “public ligand” for NK inhibitory receptors thus set the stage for subsequent exploration of its role in cancer and infectious disease. Subsequent studies revealed that HLA-G also inhibited CD8^+^ CTLs, even when the complementary inhibitory HLA-E/CD94/NKG2A axis was disrupted, indicating that HLA-G can directly suppress CTL function through distinct mechanisms (25). These early reports established the fundamental paradigm of HLA-G as a suppressor of cytotoxicity across multiple effector subsets.

Further work confirmed the breadth of HLA-G’s suppressive capacity. In xenotransplantation models, transfection of porcine endothelial cells with HLA-G1 protected them from human NK cytotoxicity (26). Similarly, melanoma cells were found to express HLA-G as a means of resisting NK-mediated killing (27). In triple-negative breast cancer, Indoleamine 2,3 – Dioxygenase 1 (IDO-1) upregulation increases HLA-G expression, thereby suppressing NK cell antitumor activity; blockade of IDO-1 can restore NK cell cytotoxicity (28). These studies led to the identification of HLA-G as a shared mechanism of immune escape across diverse settings (**Figure 1b**). Importantly, NK inhibition was shown to be dose dependent: higher levels of HLA-G expression correlated with greater resistance to NK lysis (29). Mechanistically, this suppression was independent of lipid raft integrity on target cells, indicating that HLA-G acts through receptor engagement rather than broader membrane remodeling (30). The relevance of HLA-G extends beyond NK cells to adaptive immunity. In patients with acute decompensated cirrhosis, an expanded CD4^+^HLA-G^+^ regulatory T cell population was identified and associated with poor outcomes. This non-classical population suppresses T cell function through a CTLA-4-dependent pathway and impair host defense by downregulating Th17 cytokines (31). In pleural tuberculosis, overexpression of HLA-G suppressed T cell production of IFN-γ and TNF-α, with antibody blockade restoring cytokine secretion (32). Soluble forms of HLA-G have been shown to induce apoptosis in both NK and CD8^+^ T cells, further reinforcing its ability to globally dampen cytotoxic responses (4). These diverse contexts illustrate the shared theme that HLA-G operates as a cytotoxicity regulator, positioning it as both a functional effector molecule and a marker of suppressed immune potential.

Isoform diversity adds complexity to these observations. Both HLA-G1 and HLA-G5 were found to act additively in inhibiting NK killing (33), while β2m-free HLA-G isoforms unexpectedly enhanced NK cytotoxicity and pro-inflammatory cytokine release (34). These findings suggest that HLA-G is not a uniform inhibitor of cytotoxicity, but rather a family of molecules with context-dependent effects. Distinguishing between isoforms therefore becomes essential when evaluating HLA-G as a biomarker of cytotoxic potential in clinical specimens. Another layer of complexity in HLA-G-mediated immune evasion arises from its transfer via trogocytosis (35, 36). It has been shown that NK cells can acquire HLA-G from HLA-G^+^ tumor cells, leading to impaired cytotoxicity against HLA-G^-^ melanoma targets. Importantly, blocking HLA-G restored NK cytotoxicity and reestablished their capacity to modulate other ILT2^+^ effector cells (37). Overall, HLA-G emerges as a potential immune checkpoint whose diverse roles define its capacity to regulate cytotoxic effector function, with broad implications for disease and therapy.

## Harnessing the context-dependent role of HLA-G in therapeutic approaches

Leveraging HLA-G’s regulatory properties offers a promising avenue for innovative therapies. Treatment with demethylating agents has been shown to restore its expression in previously HLA-G^-^ cell lines, while similar epigenetic interventions in human mesenchymal stem cells likewise induce significant upregulation of HLA-G (38, 39). Beyond these, hypomethylating agents (HAs) have also been shown to induce HLA-G expression in immune cells, giving rise to a distinct subset of HLA-G^+^ T cells with immunosuppressive capabilities. Notably, these HA-induced CD4^+^HLA-G^+^ T cells keep their ability to suppress other cells even under inflammatory signals and HA withdrawal (40). Transcriptomic profiling of HA-treated HLA-G^+^ cells demonstrated that immune suppressive activity on this population is driven by a unique gene expression program enriched for immune regulatory pathways (41). Furthermore, they can be robustly and reproducibly manufactured under Good Manufacturing Practice conditions while maintaining stable HLA-G expression and a favorable safety profile. This work paved the way for their clinical translation and they are currently being evaluated in a phase I/II first-in-human clinical trial, for the prevention or treatment of Graft-versus-Host-Disease (GvHD) (42, 43). Beyond pharmacological and epigenetic approaches, recent studies have begun to unravel the transcriptional network controlling HLA-G expression, offering new opportunities to identify targetable regulators. For instance, TEA domain family member 1 & 3 transcription factors (TEAD1/3) and WNT signaling were shown to critically control HLA-G transcription. TEAD1/3 transactivate HLA-G by binding to its promoter and WNT signaling inhibition is required specifically for HLA-G expression in extravillous trophoblasts (44). Recent work identified a remote enhancer essential for HLA-G expression in extravillous trophoblasts, providing a molecular explanation for its selective expression at the maternal-fetal interface and highlighting an additional layer of transcriptional regulation (45).

An alternative approach involves the use of recombinant and synthetic HLA-G molecules to directly harness their tolerogenic properties. Exogenous administration of recombinant HLA-G5 was evaluated in a rat model of small bowel transplantation, where it prolonged graft survival and attenuated acute rejection. CD8^+^ T cell accumulation was reduced, indicating a dampening of cytotoxic responses (46). A subsequent study demonstrated that synthetic HLA-G proteins inhibited T cell proliferation and NK cell cytotoxicity *in vitro* (47). Notably, HLA-G-bearing extracellular vesicles have also been investigated for their therapeutic potential. In a clinical setting, mesenchymal stromal cell-derived extracellular vesicles enriched in HLA-G, IL-10 and TGF-β were administered to a patient with severe GvHD, resulting in marked clinical improvement and a substantial reduction in inflammatory symptoms (48). Collectively, recombinant HLA-G proteins and HLA-G-bearing extracellular vesicles emerge as promising therapeutic tools to harness the molecule’s tolerogenic potential.

Conversely, in tumors the opposite strategy, namely downregulation of HLA-G, may be beneficial, as HLA-G expression has been directly implicated in tumor immune evasion and therapy resistance. For instance, in HER2^+^ breast cancer, HLA-G interaction with the NK receptor KIR2DL4 promoted resistance to trastuzumab (49). Molecular regulation of HLA-G intersects closely with cytotoxic control, and several studies have shown that its downregulation can enhance antitumor immunity. RNA-based approaches, including microRNA (miRNA), short hairpin RNA (shRNA), and small interfering RNA (siRNA) transfection, have been shown to effectively reduce HLA-G expression at both the transcript and protein levels in HLA-G^+^ cell lines, and this suppression has been linked to restored NK cytotoxicity (50–52). In renal cell carcinoma, specific miRNAs were identified that downregulate HLA-G expression, correlating with improved immune activity and clinical outcomes (53). Additional surveys of miRNAs targeting HLA-G have expanded the repertoire of potential regulators (54). More broadly, HLA-G expression in solid tumors has been consistently associated with suppression of NK and CTL activity, while in some hematological malignancies, paradoxically, HLA-G has been reported to reduce malignant B cell proliferation (55). These findings emphasize the importance of post-transcriptional regulation as a strategy to restore cytotoxic immunity in tumors, while underscoring the need for disease-specific approaches before generalizing therapeutic interventions.

In the context of CAR-T cell immunotherapy, HLA-G has been targeted by engineered CAR T cells, aiming to eliminate HLA-G-expressing tumor cells and thereby overcome its immune checkpoint function (56), and is currently under investigation in a Phase I/II clinical trial (57), among other therapeutic approaches (**Table 1**). Building on this, HLA-G-specific CAR-γδT cells have emerged as a new immunotherapeutic strategy. When further engineered to secrete bispecific T cell engagers (BiTEs) against immune checkpoints such as PD-L1, these CAR-γδT cells exhibited potent antitumor activity (58).

**Table 1:** Clinical studies for HLA-G-based immunotherapies.

Title	Phase	Study type	Type of intervention	Indications	Primary outcome
A Prospective Study of the Relevance of the HLA-G Immune Checkpoint in Cancer Immunotherapy (GEIA) (NCT04300088)	–	Observational	–	Patients with advanced solid cancer treated with anti-PD(L)1	The impact of HLA-G tumor expression (evaluated by immunohistochemistry) on tumor response rates (evaluated with iRECIST)
Study of SAR444881 Administered Alone and in Combination With Other Therapeutics in Participants With Advanced Solid Tumors (NCT04717375)	Phase I/II	Interventional	Humanized ILT2 blocking antibody	Patients with unresectable or metastatic disease who are refractory to or are not candidates for standard approved therapy	Incidence of treatment-emergent adverse events (TEAEs) dose limiting toxicities (DLT), serious adverse events and Objective Response Rate (ORR) per RECIST v1.1
TTX-080 HLA-G Antagonist in Subjects With Advanced Cancers (NCT04485013)	Phase I	Interventional	HLA-G Antagonist	Patients with advanced refractory/resistant solid malignancies including metastatic colorectal cancer (mCRC) patients.	Determine the anti-tumor activity of TTX-080 by objective response rate (complete response + partial response) for each tumor arm per RECIST 1.1
A Phase I/II Trial of UCB4594 in Participants With Advanced Cancer (NCT06380816)	Phase I/II	Interventional	Monoclonal antibody against HLA-G	Patients with advanced cancer	Recommended Phase 2 dose (RP2D) of UCB4594. Frequency of adverse events (AEs) considered at least possibly related to UCB4594
A Study of JNJ-78306358 in Participants With Advanced Stage Solid Tumors (NCT04991740)	Phase I	Interventional	BiTE (CD3/HLA-G)	Patients with advanced cancer, including renal cell carcinoma (RCC), ovarian cancer and colorectal cancer (CRC)	Number of participants with incidence of Adverse Events (AEs), severity and Dose-Limiting Toxicity (DLT)
A Safety And Efficacy Study Of HLA-G- Targeted CAR-T Cells IVS-3001 In Subjects With Previously Treated Advanced HLA-G-Positive Solid Tumors (NCT05672459)	Phase I/II	Interventional	Autologous anti-HLA-G CAR T cells	Patients with previously treated advanced HLA-G-positive solid tumors	Incidence of Adverse Events (AEs), Objective Response Rate (ORR) according to RECIST 1.1
Phase I/II study of HLA-G + induced T-regulatory cells (iG-Tregs) in patients after allogeneic hematopoietic stem cell transplantation from HLA compatible sibling/donor (EudraCT 2021-006367-26)	Phase I/II	Interventional	Pharmacologically induced HLA-G+ T regulatory cells (iG-Tregs)	Adult patients undergoing HSCT from a fully compatible donor sibling for the prevention and treatment of GvHD	Determine the safety, tolerance and maximum tolerated dose (MTD) of iG-Treg infusion to prevent GvHD

Antibody therapeutics represent another promising frontier. A bispecific T cell engager, JNJ-78306358, was recently described that simultaneously targets CD3 and HLA-G, enabling direct redirection of T cells toward HLA-G^+^ tumor cells (59). More recently, trispecific engagers incorporating PD-L1, HLA-G, and CD3 have been developed to overcome tumor heterogeneity, particularly in lung cancer (60). These approaches reflect a convergence toward multi-checkpoint targeting, acknowledging that HLA-G could be targeted in tandem with other inhibitory pathways such as PD-L1.

## Discussion

Current evidence positions HLA-G as a powerful regulator of immune homeostasis. HLA-G function extends across diverse settings from physiological tolerance in pregnancy, to post-transplant regulation. However, it has a context-dependent role representing both a therapeutic promise and a potential risk if unwanted immune suppression occurs. Taken together, a challenge moving forward is to disentangle when HLA-G serves a tolerogenic function and when it promotes immune escape.

The ability of HLA-G to drive the development of cells with immunosuppressive or hypoimmunogenic properties underlies its growing therapeutic relevance. Pharmacological modulation, such as treatment with hypomethylating agents, can induce HLA-G expression and give rise to cells with durable immunosuppressive capabilities. Noteworthy, ectopic expression of HLA-G has been employed to engineer cells with hypoimmunogenic potential, reducing their immunogenicity and resistance to NK- and T-cell-mediated rejection both *in vitro* and *in vivo* (61–63). Finally, exogenous delivery of recombinant HLA-G or HLA-G-bearing extracellular vesicles provides an additional means of modulating immune responses in a controlled and context-dependent manner. However, there are critical open questions that must be resolved. For instance, the influence of disease-specific microenvironments on the function and persistence of HLA-G^+^ cells must be clarified, and further optimization of such therapies is required-including more precise epigenetic modulation of HLA-G expression using next-generation gene editing technologies as well as careful evaluation of potential off-target effects associated with these emerging approaches. Advances in single-cell and spatial transcriptomics may shed light on how HLA-G-expressing cells alter tissue microenvironments during disease progression or remission. Moreover, the field of humanized models provides a unique opportunity to study the HLA-G mediated regulation, safety and efficacy *in vivo*.

Leveraging its extended properties in the cytotoxic context, HLA-G emerges as both a biomarker and an active effector of suppressed cytotoxicity. Its expression directly correlates with diminished NK and CTL activity, making it a valuable marker for identifying tumors and infection sites characterized by poor immune infiltration and suppressed cytotoxic potential. At the same time, mechanistic studies demonstrate that HLA-G is not a mere bystander marker but an active suppressor whose blockade restores cytotoxic function. Therapeutically, the field is moving toward combinatorial approaches that target HLA-G alongside PD-L1 and other checkpoints, using advanced platforms such as BiTEs, trispecific engagers, and CAR-T cells. Co-targeting HLA-G with the HLA-E/NKG2A axis, where co-expression occurs (64), could represent a promising future direction-particularly given that monalizumab, an NKG2A-blocking antibody, is already under clinical evaluation (NCT05903092). However, direct targeting of HLA molecules may carry safety concerns, including the risk of cytokine release syndrome (CRS) or other off-target effects, especially in the context of CAR-T cell therapy, underscoring the need for careful clinical monitoring (11).

A central challenge remains the heterogeneity of HLA-G isoforms and their divergent effects on cytotoxicity. While most isoforms inhibit NK and T cell activity, others such as β2m-free HLA-G can paradoxically enhance cytotoxicity (34). The contrasting effects of β2m-associated and β2m-free HLA-G isoforms on NK cell cytotoxicity could likely reflect their differential engagement with NK cell receptors. This suggests that patients with β2m defects may exhibit heightened responsiveness to NK cell-based immunotherapies (65, 66), as the absence of β2m coupled with compensatory HLA-G upregulation could promote NK cell activation. This diversity complicates the use of HLA-G as a straightforward biomarker and underscores the need for isoform-specific detection tools to improve stratification of patients and prediction of therapeutic response, as its context-dependent role raises the possibility that therapeutic targeting could have unintended consequences in certain contexts.

Beyond these, isoform diversity influencing receptor avidity, and receptor availability across different cell subsets, collectively modulate the extent and quality of HLA-G signaling in a context-dependent manner (67–71). HLA-G-mediated immune regulation requires the presence of its cognate receptors but the expression of HLA-G receptors (ILT2, ILT4, KIR2DL4) is not constant but varies across disease contexts (72). In certain pathophysiological states, these receptors may be downregulated (73). Moreover, ILT2 shows preferential recognition of β2m-associated HLA-G dimers, while ILT4 is capable of binding both β2m-associated and β2m-free dimers of HLA-G1 and HLA-G5 (74). Thus, future studies and clinical applications should incorporate receptor expression and avidity profiling when evaluating HLA-G as a biomarker or therapeutic agent.

Technical variability further complicates interpretation: antibody clones differ in isoform recognition (e.g. 4H84 detects all denatured isoforms, whereas MEM-G/9 preferentially bind G1/G5), and soluble HLA-G measurement depends on assay type, sample processing and handling. Therefore, studies should at minimum report the assay used, antibody clone, β2m-dependence or isoform specificity, membrane versus soluble form, cut-off criteria and internal controls.

Looking forward, key priorities include delineating the isoform-specific mechanisms of cytotoxic regulation, developing approaches such as targeted gene editing and pharmacological interventions to modulate HLA-G expression, and integrating HLA-G blockade into multimodal immunotherapy regimens (**Figures 1c, d**). Its historical role in maternal-fetal tolerance may thus find a new counterpart in tumor immunology and infectious disease, where reactivating cytotoxic immunity remains a major challenge. Collectively, these insights emphasize that HLA-G is not a static molecule but an adaptable immune checkpoint shaped by context, with the potential to evolve from a niche tolerogenic mediator into a key target in next-generation immunotherapies. However, further research is required to clarify its full clinical relevance, and a deeper understanding of its biology will be critical to ensure the safe and effective translation of HLA-G-based interventions. Nonetheless, the growing interest in HLA-G highlights its expanding potential across a broad range of immune-mediated conditions.
